# Phase 1 dose-ranging study of ezatiostat hydrochloride in combination with lenalidomide in patients with non-deletion (5q) low to intermediate-1 risk myelodysplastic syndrome (MDS)

**DOI:** 10.1186/1756-8722-5-18

**Published:** 2012-04-30

**Authors:** Azra Raza, Naomi Galili, Deborah Mulford, Scott E Smith, Gail L Brown, David P Steensma, Roger M Lyons, Ralph Boccia, Mikkael A Sekeres, Guillermo Garcia-Manero, Ruben A Mesa

**Affiliations:** 1 Columbia University Medical Center, New York, NY, USA; 2 University of Rochester, Rochester, NY, USA; 3 Cardinal Bernardin Cancer Center, Loyola University Chicago Medical Center, Maywood, IL, USA; 4 Telik, Inc., Palo Alto, CA, USA; 5 Dana-Farber Cancer Institute, Boston, MA, USA; 6 Cancer Care Centers South Texas/US Oncology, San Antonio, TX, USA; 7 Center for Cancer & Blood Disorders, Bethesda, MD, USA; 8 Hematologic Oncology & Blood Disorders, Cleveland Clinic Taussig Cancer Institute, Cleveland, OH, USA; 9 Leukemia, The University of Texas MD Anderson Cancer Center, Houston, TX, USA; 10 Mayo Clinic, Scottsdale, AZ, USA

**Keywords:** MDS, Ezatiostat, Lenalidomide, Phase 1, Non-deletion (5q)

## Abstract

**Background:**

Ezatiostat, a glutathione S-transferase P1-1 inhibitor, promotes the maturation of hematopoietic progenitors and induces apoptosis in cancer cells.

**Results:**

Ezatiostat was administered to 19 patients with non-deletion(5q) myelodysplastic syndrome (MDS) at one of two doses (2000 mg or 2500 mg/day) in combination with 10 mg of lenalidomide on days 1–21 of a 28-day cycle. No unexpected toxicities occurred and the incidence and severity of adverse events (AEs) were consistent with that expected for each drug alone. The most common non-hematologic AEs related to ezatiostat in combination with lenalidomide were mostly grade 1 and 2 fatigue, anorexia, nausea, diarrhea, and vomiting; hematologic AEs due to lenalidomide were thrombocytopenia, neutropenia, and anemia. One of 4 evaluable patients (25%) in the 2500/10 mg dose group experienced an erythroid hematologic improvement (HI-E) response by 2006 MDS International Working Group (IWG) criteria. Four of 10 evaluable patients (40%) in the 2000 mg/10 mg dose group experienced an HI-E response. Three of 7 (43%) red blood cell (RBC) transfusion-dependent patients became RBC transfusion independent, including one patient for whom prior lenalidomide monotherapy was ineffective. Three of 5 (60%) thrombocytopenic patients had an HI-platelet (HI-P) response. Bilineage HI-E and HI-P responses occurred in 3 of 5 (60%), 1 of 3 with HI-E and HI-N (33%), and 1 of 3 with HI-N and HI-P (33%). One of 3 patients (33%) with pancytopenia experienced a complete trilineage response. All multilineage responses were observed in the 2000/10 mg doses recommended for future studies.

**Conclusions:**

The tolerability and activity profile of ezatiostat co-administered with lenalidomide supports the further development of ezatiostat in combination with lenalidomide in MDS and also encourages studies of this combination in other hematologic malignancies where lenalidomide is active.

**Trial registration:**

Clinicaltrials.gov: NCT01062152

## Background

Myelodysplastic syndromes (MDS) represent a diverse group of acquired hematopoietic stem cell disorders characterized by ineffective hematopoiesis and a variable risk of transformation to acute myeloid leukemia (AML) [1]. Prognostically, MDS has been divided into two main groups according to whether there is a higher or lower risk of developing leukemia [2]. At diagnosis, two-thirds of MDS patients present with lower-risk disease. A select group of lower-risk patients with clonally restricted deletion of the long arm of chromosome 5 (del[5q]) and transfusion-dependent anemia respond to lenalidomide, but lenalidomide is less effective in the majority of patients with MDS who lack del(5q) [3]. While the hypomethylating drugs azacitidine and decitabine are approved by the Food and Drug Administration (FDA) for treatment of patients with a broad range of MDS subtypes, the benefit of these agents for lower-risk patients is not clearly established. Other therapies that have been studied for treatment of MDS include hematopoietic growth factors, immunosuppressive therapy, various biologic response modifiers, and traditional cytotoxic chemotherapies; however, there remains a major need for new treatment options.

Ezatiostat hydrochloride (Telintra), a glutathione-analog reversible inhibitor of the enzyme glutathione S-transferase P1-1 (GSTP1-1), is being developed for the treatment of cytopenias associated with International Prognostic Scoring System (IPSS) Low or Intermediate-1 risk MDS. Ezatiostat facilitates dissociation of GSTP1-1 from jun-N-terminal kinase (JNK), leading to activation of JNK and the subsequent promotion of growth and maturation of normal multilineage hematopoietic progenitors, while promoting apoptosis in human leukemia blasts [4,5]. Recent reports have shown that GSTP1-1 may be an important mediator of signaling in hematopoietic cells [4]. In addition, the ability of ezatiostat to activate the caspase-dependent apoptotic pathway may inhibit the emergence of malignant clones, while ezatiostat's ability to increase reactive oxygen species in dysplastic cells may contribute to apoptosis in dysplastic cells [6]. These mechanistic features provide an attractive profile for modulating the biology in MDS.

Recently, a randomized multicenter phase 2 study of ezatiostat was conducted in 89 heavily pretreated patients with IPSS Low or Intermediate-1 risk MDS on two extended dose schedules. In dose schedule 1, patients received ezatiostat at 1500 mg orally (PO) twice daily (b.i.d.) for 2 weeks followed by a 1-week rest period in a 3-week treatment cycle and in dose schedule 2, patients received ezatiostat at 1000 mg PO b.i.d. for 3 weeks followed by a 1-week rest period in a 4-week treatment cycle. Multilineage hematologic responses were seen and 29% of RBC-transfusion-dependent patients experienced an erythroid response. Ezatiostat had a very favorable tolerability profile, with gastrointestinal side effects being the predominant adverse event (AE); however, these were mainly restricted to grades 1 and 2, confirming the results of the prior studies. The transfusion independence rate was higher (40%) in the subset of patients previously treated with lenalidomide (and no prior hypomethylating agents), suggesting a potential role for combining the two drugs. Finally, since ezatiostat does not have a myelosuppressive effect, it may act as a cytoprotective agent when used with lenalidomide, which is known to be significantly myelotoxic. The doses selected for this combination therapy study were selected based on the results from the phase 2 single-agent ezatiostat study. [7] The lenalidomide dose selected is based upon the approved dose of lenalidomide for the treatment of MDS. [8] The current phase 1 study was therefore conducted to determine the safety and efficacy of ezatiostat in combination with lenalidomide in IPSS Low and Intermediate-1 risk MDS patients.

## Materials and methods

This study was conducted in accordance with the International Conference on Harmonization and Good Clinical Practice standards. Institutional review board approval was obtained from all participating institutions. All patients provided written informed consent before study participation.

### Patient population

Eligible patients were 18 years or older, with histologically confirmed diagnoses of non-del(5q) MDS, Low or Intermediate-1 IPSS risk group, and Eastern Cooperative Oncology Group performance status (ECOG PS) of 0 or 1. The following World Health Organization (WHO) classification MDS subtypes were included: refractory anemia (RA), refractory nemia with excess blasts type I (RAEB-I), refractory anemia with ring sideroblasts (RARS), refractory cytopenia with multilineage dysplasia (RCMD), refractory cytopenia with multilineage dysplasia with ringed sideroblasts (RCMD-RS), MDS-unclassified (MDS-U), and MDS/myeloproliferative neoplasm-unclassified (MDS/MPN-U) without leukocytosis. Patients were required to have adequate hepatic and renal function. Inclusion criteria allowed multilineage cytopenias with assessment of response by International Working Group (IWG) 2006 criteria [9], <10% marrow blasts, and ineligibility or unwillingness to undergo allogeneic stem cell transplantation. Patients were excluded for prior allogeneic bone marrow transplantation, a history of IPSS higher-risk MDS or of AML [10,11], proliferative chronic myelomonocytic leukemia, use of oral corticosteroids at a dose exceeding 10 mg daily, history of hepatitis B/C or human immunodeficiency virus, or an active infection requiring intravenous antibiotics. Patients were not allowed to receive hematopoietic growth factors while on study, and a 4-week washout period for growth factors and all prior MDS treatments was required before study enrollment.

### Study design

This was a multicenter phase 1 dose-ranging study evaluating ezatiostat in combination with lenalidomide in patients with non-del(5q) IPSS Low or Intermediate-1 risk MDS. The hematologic improvement-erythroid (HI-E), HI-Neutrophil (HI-N), and HI-Platelet (HI-P) rates by IWG 2006 criteria and safety of each treatment group were evaluated to select the optimal dose of ezatiostat in combination with lenalidomide for future studies. Ezatiostat was given at a starting dose of 2000 mg total daily in divided doses (1000 mg PO twice daily) in combination with lenalidomide at a starting dose of 10 mg PO once daily on days 1–21 of a 28-day cycle. In each stage, three to six patients in a standard 3 + 3 design were treated before escalation to the next higher dose level; stage 2 was an ezatiostat/lenalidomide 2500 mg/10 mg dose level. Once dose-escalation was complete, the maximum tolerated dose (MTD) cohort was expanded by an additional 10 patients in stage 2. The primary objectives of this study were to establish the MTD of ezatiostat in combination with lenalidomide as well as to determine the safety of the combination. The secondary objectives of this study were to assess efficacy by determining rates of HI-E, HI-N, and HI-P. Patients were treated until MDS disease progression, lack of MDS response, unacceptable toxicities, or patient withdrawal from the study.

Red blood cell (RBC) transfusion guidelines were provided in the protocol and RBC transfusions were to be given for a hemoglobin (Hgb) level < 9.0 g/dL. All treated patients were monitored for safety and efficacy with physical and laboratory examinations and hematologic response assessments (IWG 2006) were evaluated every two cycles [12]. Adverse events were graded in accordance with the National Cancer Institute − Common Toxicity Criteria for Adverse Events, Version 3.0 (NCI − CTCAE, v3.0; Bethesda, MD) [13].

### Drug formulation

Ezatiostat is formulated as 500 mg tablets. Each tablet contains ezatiostat hydrochloride with the following excipients: mannitol, croscarmellose sodium, hypromellose, magnesium stearate, and Opadry Clear. Opradry Clear is a mixture of hypromellose and polyethylene glycol 400.

### Assessments

On day 1 of each treatment cycle, a physical examination and laboratory assessments (complete blood count [CBC] with differential and serum chemistry profile) were obtained, use of concomitant medication(s) was documented, AEs were assessed, and RBC transfusion requirements were documented for the preceding 8-week baseline period. CBC with differential and platelet count was obtained weekly.

### Dose modifications

Patients who experienced a treatment-related non-hematologic AE grade 3 or higher had treatment delayed for up to 3 weeks or until recovery to grade 1 or baseline, and subsequent treatment resumed at a dose reduced by the amount of ezatiostat of 500 mg (1 tablet) per day. If recovery did not occur after a delay of 21 days, treatment was discontinued and patients were followed until resolution of the AE. Patients were dosed at a starting dose of lenalidomide (Revlimid®) at 10 mg using commercial supply. If a patient experienced prolonged thrombocytopenia and neutropenia, that patient's lenalidomide dosage was adjusted per the lenalidomide package insert. Since lenalidomide is excreted by the kidney, dose adjustments for renal impairment were made in accordance with the package insert.

### Statistical analysis

Assuming the population incidence of an adverse event is 10% or higher, a sample size of 16 patients had a probability of 82% of observing at least one adverse event. All enrolled patients were included in the intent-to-treat (ITT) population. A secondary endpoint of the study was to determine the HI response rate in the erythroid cell line (HI-E) by the IWG MDS criteria (2006) in the ITT and efficacy-evaluable (EE) populations. All enrolled patients who received any amount of ezatiostat in combination with lenalidomide treatment were included in the safety analysis. Patients who received at least two cycles of ezatiostat in combination with lenalidomide treatment and for whom an HI response assessment was completed were included in the EE population. All investigator-determined responses were independently reviewed and confirmed. Efficacy analyses were performed in the ITT and the EE populations by the two dose groups. Exact binomial 95% confidence intervals were provided for response rates. Duration of response was estimated by Kaplan-Meier method. HI-E is defined in the IWG 2006 criteria as RBC-transfusion-dependent patients who experience a clinically significant transfusion reduction by ≥ 4 units from baseline over 8 weeks after the initiation of study treatments (given for Hgb < 9.0 g/dL); RBC transfusion independence is defined as no RBC transfusions required over an 8-week period. Time to HI-E response was calculated from the day of the initiation of study treatment to the end day of the 8-week period when an HI-E was declared. Time to RBC-transfusion independence was calculated from the day of the initiation of study treatment to the end day of an 8-week period free of transfusions. Duration of HI-E response was calculated from the end day of the 8-week period when an HI-E was declared to the first day when an HI-E criterion was no longer met. Duration of RBC-transfusion independence was calculated from the last day after an 8-week period free of transfusions to the date an RBC transfusion was required. These duration-of-response definitions pertained only when transfusion reduction or independence had been sustained beyond 8 weeks, per IWG 2006 criteria.

The safety of ezatiostat in combination with lenalidomide was evaluated by determining the frequency, severity (NCI − CTCAE v3.0), and causal relationship(s) of AEs that occurred during the treatment period and follow-up period of 30 days from the last administration of study drug treatment(s). The incidence and percentage of AEs related to study treatment, as judged by investigators, was reported by total daily dose levels of ezatiostat in combination with lenalidomide.

## Results and discussion

### Patient demographics and MDS disease characteristics

Nineteen patients, 14 men (74%) and five women (26%), median age 75 years (range 57–82), were treated in two dose groups at nine centers in the United States between June 1, 2010, and December 30, 2011. Patient demographics and MDS disease characteristics are shown in Table 1. The majority of enrolled patients were in the IPSS Intermediate-1 risk category 14 (74%). WHO subtypes were a typical distribution for non-del(5q) MDS population. Thirteen (68%) patients were RBC-transfusion dependent and the median transfusion requirements were 6 units over an 8-week period (range 4–10). Ten (53%) patients had bilineage or trilineage cytopenia.

**Table 1:** Patient Demographics and MDS Disease Characteristics

**Age (years)**	
Median (range)	75 (57−82)
**Age (years)**	**N (%)**
< 65 years	5 (26)
≥ 65 years	14 (74)
**Gender**	
Male	14 (74)
Female	5 (26)
**ECOG Performance Status**	
0	13 (68)
1	6 (32)
**IPSS Classification**	
Low Risk	5 (26)
Intermediate-1 Risk	14 (74)
**Baseline Cytogenetics**	
Normal	14 (74)
Abnormal	4 (21)
Unknown	1 (5)
**Transfusion Dependency**	
RBC Transfusion Dependent	13 (68)
Median (Range)	6 (4−10)
Platelet Transfusion Dependent	2 (11)
**WHO Classification**	
RA	4 (21)
RAEB-1	2 (11)
RCMD	4 (21)
RCMD-RS	5 (26)
MDS-U	3 (16)
MDS/MPD-U	1 (5)
**Cell Lineage Cytopenia**	
Unilineage Cytopenia	9 (47)
Bilineage Cytopenia	6 (32)
Trilineage Cytopenia	4 (21)
**Baseline Cytopenia(s)**	
Anemia only	9 (47)
Anemia + Neutropenia	1 (5)
Anemia + Thrombocytopenia	5 (26)
Anemia + Neutropenia + Thrombocytopenia	4 (21)
**Time from MDS Diagnosis (years)**	
Median (range)	.9 (.10 − 2.5)

Abbreviations: *ECOG* Eastern Cooperative Oncology Group; *IPSS* International Prognostic Scoring System; *RBC* red blood cell; *RA* refractory anemia; *RAEB-1* RA with excess blasts, type 1; *RCMD* refractory cytopenia with multilineage dysplasia; *RCMD-RS* RCMD with ringed sideroblasts; *MDS-U* myelodysplastic syndrome-unclassified; *MDS/MPN-U* MDS/myeloproliferative neoplasm-unclassified.

Prior therapies are shown in Table 2. Ten (53%) patients had received prior erythropoietin therapy. Three (16%) patients had a history of failing to respond to prior lenalidomide monotherapy.

**Table 2:** Prior MDS Therapies

**Treated Population**	**(N = 19)**
Prior Therapies − Median (Range)	1 (0–6)
	**N (%)**
Chemotherapy	2 (11)
Immunotherapy	1 (5)
G-CSF	3 (16)
Investigational Agent/Drug	2 (11)
Erythropoietin	10 (53)
Steroids	3 (16)
Vitamins	2 (11)
Lenalidomide	3 (16)

Abbreviations: *G-CSF* granulocyte colony stimulating factor.

### Ezatiostat in combination with lenalidomide treatments administration

At the time of submission of this report, four patients are continuing on extended therapy at the recommendation of their investigator, due to continuing clinical benefit. A summary of treatment administration is shown in Table 3. Lenalidomide dose reductions were infrequent, with only 9% of all cycles requiring dose reductions and 13% requiring dose delays. Dose reductions were due to nausea (4 cycles), diarrhea (3 cycles), vomiting (4 cycles), neutropenia (1 cycle), anxiety (2 cycles), and gastritis or acute renal insufficiency (1 cycle). Dose delays were most frequently due to thrombocytopenia and neutropenia. Two of six patients reported dose-limiting toxicities, which consisted of grade 3 diarrhea and grade 3 rash; both were in the ezatiostat/lenalidomide 2500 mg/10 mg dose group. The ezatiostat/lenalidomide 2000 mg/10 mg dose level was therefore selected as the MTD for enrollment of 10 additional patients in stage 2. A total of 13 patients were treated at the 2000 mg/10 mg combination dose level.

**Table 3:** Treatment Administration

	**Dose Group 1**	**Dose Group 2**	
**Safety Population**	**2000/10 mg**	**2500/10 mg**	**Total**
**No. of Patients**	13	6	19
**Total No. of Cycles Administered**	56	23	79
**Median No. of Cycles per Patient (Range)**	4 (1−14)	3.5 (1−7)	4 (1−14)
**No. of Cycles with Dose Reduction (% of total cycles)**	4 (7)	3 (13)	7 (9)
**No. of Cycles with Dose Delay (% of total cycles)**	7 (13)	3 (13)	10 (13)

### Safety

Details of ezatiostat plus lenalidomide combination-related adverse events by grade and combined dose levels are presented in Table 4. No unexpected toxicities occurred and the incidence and severity of AEs were consistent with that expected for each drug alone. The most common combination treatment-related non-hematologic AEs in the combined dose levels were fatigue, anorexia, nausea, diarrhea, and vomiting. There was one grade 4 event of hypersensitivity. The most common hematologic treatment-related AEs were thrombocytopenia, neutropenia, and anemia, which are common with lenalidomide and were rare in prior ezatiostat monotherapy studies. There were two grade 3 febrile neutropenia events. Ezatiostat in combination with lenalidomide AEs by dose level and frequency are presented in Table 5.

**Table 4:** Adverse Events Related to Ezatiostat in Combination with Lenalidomide for Both Dose Groups Combined

**NCI-CTCAE v3.0 Maximum Toxicity Grade (N = 19)**					
**Adverse Event (Preferred Term)**	**Grade 1**	**Grade 2**	**Grade 3**	**Grade 4**	**Total**
		**n (%)**	**n (%)**	**n (%)**	**n (%)**	**n (%)**
**Hematologic (All Patients)**					
Thrombocytopenia	1 (5)	1 (5)	4 (21)	3 (16)	9 (47)
Neutropenia	0	2 (11)	2 (11)	2 (11)	6 (32)
Anemia	0	0	3 (16)	1 (5)	4 (21)
Febrile Neutropenia	0	0	2 (11)	0	2 (11)
**Non-Hematologic (≥ 5% of Patients)**					
Diarrhoea	5 (26)	3 (16)	3 (16)	0	11 (58)
Nausea	7 (37)	3 (16)	1 (5)	0	11 (58)
Vomiting	6 (32)	4 (21)	1 (5)	0	11 (58)
Fatigue	1 (5)	5 (26)	0	0	6 (32)
Skin Odour Abnormal	3 (16)	1 (5)	0	0	4 (21)
Abdominal Pain Upper	2 (11)	1 (5)	0	0	3 (16)
Anorexia	2 (11)	1 (5)	0	0	3 (16)
Rash	0	1 (5)	2 (11)	0	3 (16)
Constipation	2 (11)	0	0	0	2 (11)
Flatulence	1 (5)	1 (5)	0	0	2 (11)
Muscle Spasms	1 (5)	0	1 (5)	0	2 (11)
Muscular Weakness	0	1 (5)	1 (5)	0	2 (11)
Oedema Peripheral	1 (5)	1 (5)	0	0	2 (11)

Abbreviations: *NCI-CTCAE v3.0* National Cancer Institute − Common Toxicity Criteria for Adverse Events, Version 3.0.

**Table 5:** Adverse Events Related to Ezatiostat in Combination with Lenalidomide by Dose Level

**Adverse Event (Preferred Term)**	**Total Daily Starting Doses**	
	**Dose Group 1**	**Dose Group 2**
	**2000/10 mg/day**	**2500/10 mg/day**
	**(N = 13)**	**(N = 6)**
	**n (%)**	**n (%)**
**Hematologic (All Patients)**		
Thrombocytopenia	7 (54)	2 (33)
Neutropenia	4 (31)	2 (33)
Anaemia	3 (23)	1 (17)
Febrile Neutropenia	2 (15)	0
**Non-Hematologic (≥ 10% of Patients)**		
Vomiting	9 (69)	2 (33)
Diarrhea	8 (62)	3 (50)
Nausea	7 (54)	4 (67)
Fatigue	4 (31)	2 (33)
Abdominal Pain Upper	2 (15)	1 (17)
Constipation	2 (15)	0
Flatulence	2 (15)	0
Anorexia	2 (15)	1 (17)
Muscle Spasms	2 (15)	0
Muscle Weakness	2 (15)	0
Skin Odour Abnormal	3 (23)	1 (17)
Gastritis Erosive	0	1 (17)
Chills	0	1 (17)
Oedema Peripheral	1 (8)	1 (17)
Hypersensitivity	0	1 (17)
Occult Blood Positive	0	1 (17)
Gout	0	1 (17)
Flank Pain	0	1 (17)
Dysgeusia	0	1 (17)
Renal Failure Acute	0	1 (17)
Epitaxis	0	1 (17)
Rash	1 (8)	2 (33)
Night Sweats	0	1 (17)
Swelling Face	0	1 (17)

There were 16 combination treatment serious adverse events (SAEs). Six events were assessed by the principal investigator as related or possibly related to ezatiostat in combination with lenalidomide treatment, including one event each of rash, acute renal insufficiency, anemia with guaiac positive stools, febrile neutropenia, syncope, and erosive gastritis. These events resolved without sequelae.

### Efficacy

Nineteen patients were in the treated population, and 14 of 19 patients were in the EE population. Of the 13 of 19 patients who were RBC-transfusion-dependent at enrollment, 10 were treated at the 2000/10 mg dose level and three treated at the 2500/10 mg dose level.

The majority of HI-E responders and all RBC-transfusion-dependent patients who responded to combination therapy were in the 2000/10 mg dose group. One of four evaluable patients (25%) in the 2500/10 mg dose group experienced an HI-E response (IWG 2006). Four of 10 evaluable patients (40%) in the 2000 mg/10 mg dose group experienced an HI-E response. Three of seven evaluable RBC-transfusion-dependent patients achieved RBC-transfusion independence (43%; 95% CI, 0.8 − 90.6%), including one RBC-transfusion-independent patient who did not previously respond to lenalidomide. Median duration of transfusion independence has not yet been reached.

All multilineage responses were observed at the 2000/10 mg dose level. In three of five (60%; 95% CI 14.7%−94.7%) evaluable thrombocytopenic patients, an HI-P response was observed, including one patient with platelet-transfusion dependence who achieved platelet-transfusion independence. One of three patients with neutropenia achieved an HI-N response, for an HI-N rate in the eligible group of 33.3% (95% CI, 0.8−90.6%). Bilineage responses included HI-E and HI-P responses in three of five patients with both anemia and thrombocytopenia (60%; 95% CI, 14.7%−94.7%); HI-E and HI-N responses in one of three patients with both anemia and neutropenia (33.3%; 95% CI, 0.8%−90.6%); and HI-N and HI-P responses in one of three patients with both neutropenia and thrombocytopenia (33.3%; 95% CI, 0.8%−90.6%). One of three patients with trilineage cytopenia had a complete response of all three cell lineages (33.3%; 95% CI, 0.8%−90.6%) (Table 6). One of three evaluable patients (33.3%) who had failed prior lenalidomide therapy and was RBC-transfusion-dependent became RBC-transfusion-independent. One RBC- and platelet-transfusion-dependent patient who had a poor response to prior anti-thymocyte-globulin treatment achieved complete RBC- and platelet-transfusion independence.

**Table 6:** Efficacy

	**Dose Group 1**	**Dose Group 2**
**Intent-to-Treat (ITT) Population**	**2000/ 10 mg**	**2500/ 10 mg**
HI–E (%) (95% CI)	**(N = 13)**	**(N = 6)**
	4 (30.8)	1 (16.7)
	(9.1−61.4)	(0.4−64.1)
HI–N (%) (95% CI)	**(N = 3)**	**(N = 2)**
	1 (33.3)	0
	(0.8−90.6)	(0−84.2)
HI–P (%) (95% CI)	**(N = 5)**	**(N = 4)**
	3 (60)	0
	(14.7−94.7)	(0−60.2)
Platelet Transfusion Dependent (%)	**(N = 1)**	**(N = 1)**
Platelet Transfusion Independent (%)	1 (100)	0 (0)
Bilineage (HI-E and HI-N) (%) (95% CI)	**(N = 3)**	**(N = 2)**
	1 (33.3)	0
	(0.8−90.6)	(0−84.2)
Bilineage (HI-E and HI-P) (%) (95% CI)	**(N = 5)**	**(N = 4)**
	3 (60)	0
	(14.7−94.7)	(0−60.2)
Bilineage (HI-N and HI-P) (%) (95% CI)	**(N = 3)**	**(N = 1)**
	1 (33.3)	0 (0)
	(0.8−90.6)	(0−97.5)
Trilineage (HI-E, HI-N, and HI-P) (%) (95% CI)	**(N = 3)**	**(N = 1)**
	1 (33.3)	0
	(0.8−90.6)	(0−97.5)
No. of RBC Transfusion Dependent Patients (%)	10 (76.9)	3 (50)
RBC Transfusion Independence (%) (95% CI)	3 (30)	0
	(6.7−65.2)	(0−70.8)
**Efficacy Evaluable (EE) Population**		
HI–E (%) (95% CI)	**(N = 10)**	**(N = 4)**
	4 (40)	1 (25)
	(12.2−73.8)	(0.6−80.6)
HI–N (%) (95% CI)	**(N = 3)**	**(N = 2)**
	1 (33.3)	0 (0)
	(0.8−90.6)	(0−84.2)
HI–P (%) (95% CI)	**(N = 5)**	**(N = 2)**
	3 (60)	0 (0)
	(14.7−94.7)	(0−84.2)
Platelet Transfusion Dependent	**(N = 1)**	**(N = 0)**
Platelet Transfusion Independent (%)	1 (100)	0 (0)
Bilineage (HI-E and HI-N) (%) (95% CI)	**(N = 3)**	**(N = 2)**
	1 (33.3)	0 (0)
	(0.8−90.6)	(0−84.2)
Bilineage (HI-E and HI-P) (%) (95% CI)	**(N = 5)**	**(N = 2)**
	3 (60)	0 (0)
	(14.7−94.7)	(0−84.2)
Bilineage (HI-N and HI-P) (%) (95% CI)	**(N = 3)**	**(N = 1)**
	1 (33.3)	0 (0)
	(0.8−90.6)	(0−97.5)
Trilineage (HI-E, HI-N, and HI-P) (%) (95% CI)	**(N = 3)**	**(N = 1)**
	1 (33.3)	0 (0)
	(0.8−90.6)	(0−97.5)
No. of RBC Transfusion Dependent Patients (%)	7 (70)	2 (50)
RBC Transfusion Independence (%) (95% CI)	3 (42.9)	0
	(9.9−81.6)	(0−84.2)

Abbreviations: *HI-E* hematologic improvement-erythroid; *HI-N* hematologic improvement-neutrophil; *HI-P* hematologic improvement-platelet; *CI* confidence interval; *RBC* red blood cell.

## Discussion

Ezatiostat is the first GSTP1-1 inhibitor shown to cause clinically significant reductions in RBC and platelet transfusions, including transfusion independence, as well as trilineage hematologic improvement—HI-E, HI-N and HI-P—in single-agent monotherapy trials in patients with IPSS Low or Intermediate-1 risk MDS [7,14,15], thereby providing a unique profile of activity in MDS.

Since myelosuppression is a side effect common to many of the available drugs for MDS, which exacerbates disease-related cytopenias, the development of effective combination chemotherapy regimens for the treatment of lower risk MDS has been a challenge. Ezatiostat, with its novel mechanism of action and its non-myelosuppressive single-agent activity, is a drug candidate for combining with lenalidomide, with the possibility of improving outcomes in these patients. The optimal dosing for the combination regimen in this phase 1 trial was determined to be ezatiostat at 2000 mg daily combined with the standard 10 mg dose of lenalidomide days 1–21 in a 28-day cycle. The combination with lenalidomide and ezatiostat showed promising hematopoietic-promoting activity in non-del(5q) lower-risk MDS patients and induced RBC-transfusion independence in patients who were RBC-transfusion dependent. The combination also induced platelet-transfusion independence in a patient who was platelet-transfusion-dependent, an effect not typically seen with lenalidomide monotherapy [16]. Trilineage and bilineage responses were also observed, as seen previously with single-agent ezatiostat; again, multilineage responses are uncommon with lenalidomide monotherapy, which is most effective with respect to the erythroid lineage. Interestingly, a patient who had failed to respond to single-agent lenalidomide subsequently responded to the combination of ezatiostat plus lenalidomide. This observation may warrant further study.

Currently, ezatiostat is being evaluated in two phase 2 studies—a 150-patient clinical study in lenalidomide-refractory or -resistant del(5q) MDS patients, and a 140-patient phase 2b clinical study in non-del(5q) MDS patients. In addition, the results of a preliminary genomic study intended to identify genomic markers that predict likelihood of response to ezatiostat was recently reported [17]. The genomic marker results may suggest the potential to develop a diagnostic test to screen MDS patients most likely to respond to ezatiostat and to utilize this test in a future trial to select patients for study.

## Conclusions

The tolerability and activity profile of ezatiostat co-administered with lenalidomide suggests that this combination warrants further evaluation in future studies of patients with IPSS Low or Intermediate-1 risk non-del(5q) MDS and also encourages studies of this combination in other hematologic malignancies where lenalidomide is active.

## Competing interests

Dr. Brown is an employee of Telik, Inc., is fully compensated and holds stock in the company. Dr. Sekeres serves on Celgene's and Amgen's advisory boards. Dr. Mulford has served on Celgene's speaker's bureau. The other investigators report no relevant conflicts of interest other than research support to their institutions from Telik, Inc. for the study conduct.

## Authors’ contributions

AR, NG, and GB designed the research protocol; AR, NG, DM, SES, DPS, RML, RB, MAS, GGM, and RAM were involved in treating patients and collecting data; AR, NG, and GB wrote the paper with contributions from the other authors. All authors read and approved the final manuscript.
